# Increased circulating obestatin in patients with chronic obstructive pulmonary disease

**DOI:** 10.1186/2049-6958-9-5

**Published:** 2014-01-28

**Authors:** Yi Lei, Yasha Liang, Yifan Chen, Xiaojing Liu, Xiaoyang Liao, Fengming Luo

**Affiliations:** 1 Department of Family Medicine, West China Hospital, Sichuan University, 37 GuoXue Xiang, Chengdu 610041, China; 2 Department of Respiratory Disease, West China Hospital, Sichuan University, 37 GuoXue Xiang, Chengdu 610041, China

**Keywords:** COPD, Inflammation, Nutrition, Obestatin

## Abstract

**Background:**

Some peptides, which regulate the metabolic balance, are thought to play important roles in nutritional disorders and systemic inflammation in COPD. Treatment of rats with obestatin decreased body-weight gain. Obestatin was also found to be correlated with inflammation in rheumatoid arthritis. The aims of this study were to investigate the level of circulating obestatin in COPD and to analyze the relationship among obestatin and nutritional status, and systemic inflammation.

**Methods:**

32 COPD patients with BMI less than 20 kg/m^2^ and 22 normal controls were included. Body composition was estimated using “foot-to-foot” BIA technology. Circulating obestatin was determined with enzyme-linked immunosorbent assay. Pulmonary function, TNF-α and C reactive protein were also measured.

**Results:**

The level of circulating obestatin was higher in COPD with underweight than that in normal control (5562.75 ± 3435.43 pg/ml in COPD, 3663.90 ± 2313.95 pg/ml in controls, p = 0.028). BMI, Waist circumference, hip circumference, bodyFAT and FAT% in COPD group were lower than those in normal control. Positive correlation was found among circulating C reactive protein, TNF-α and obestatin. There was no significant correlation among BMI, pulmonary function and obestatin.

**Conclusions:**

This study shows that circulating obestatin is higher in underweight COPD patients, and positively correlated to systemic inflammation, but not to nutritional status.

## Background

Disorders of nutritional status are regarded as common phenomena resulting from chronic obstructive pulmonary disease (COPD) [1]. Malnutrition is a major factor that increases mortality in COPD, whose frequency was reported between 20% and 40% [1,2]. Moreover, poor nutritional status increased the risk of acute exacerbation, which results in decreased lung function and life quality of the patients [2,3]. Thus, understanding the mechanism of malnutrition in COPD may be helpful to manage this disease. However, the mechanism of COPD related malnutrition largely remains unknown at present. Although a previous research showed that systemic inflammation is one of the main factors leading to malnutrition in COPD [4], understanding how the systemic inflammation regulates malnutrition in COPD is unclear. Some studies reported that digestive tract cells or adipocytes secrete peptides regulating the metabolic balance and inflammation process, and thus play important roles in the nutrition disorder in COPD [5,6]. However, previously only a few of these peptides have been studied in COPD.

Obestatin is a putative hormone secreted by the cells of the stomach and small intestine of several mammals including humans. The gene that encodes obestatin also encodes ghrelin, a peptide hormone that increases appetite [7]. The precursor protein breaks into two peptides, ghrelin and obestatin [7]. Ghrelin increases the food intake and decreases energy expenditure, and thus causes weight gain. In contrast to ghrelin, obestatin acts as an anorexic hormone by decreasing food intake, gastric emptying activities, and jejunal motility, thus, leading to body-weight loss [7]. Although circulating ghrelin was reported to take a part in the malnutrition in COPD [8,9], the role of obestatin in COPD and its relationship with COPD nutritional status remain unknown.

Obestatin was also reported to modulate the inflammation process in some diseases. Kellokoski et al. [10] reported that obestatin decreased vascular cell adhesion molecule-1 (VCAM-1) expression in the presence of tumor necrosis factor-α (TNF-α). Although there was no significant difference in the level of obestatin between normal control and rheumatoid arthritis (RA), a disease with systemic inflammation, obestatin was found to be higher in RA than in Behçet’s disease (BD), the other chronic inflammation disease. Furthermore, the level of obestatin in other diseases with systemic inflammation has not been investigated yet. In addition, obestatin was found to be positively correlated with erythrocyte sedimentation rate in the RA group, the level of IL-6 in the BD group, and with the level of TNF-α in the control group. Whether obestatin correlates with inflammatory markers in COPD remains unknown.

Therefore, the aim of this study was to investigate the relationship between the level of plasma obestatin and systemic inflammation in the malnourished COPD patients.

## Methods

### Patients

This study was approved by the medical ethics committee of the West China Hospital, Sichuan University. All subjects signed consent for taking part into this study. 2,000 people in 2 communities were screened for COPD according to the Global Initiative for Chronic Obstructive Lung Disease (GOLD) criteria [11]. Patients that had known conditions which may affect plasma obestatin levels such as gastrointestinal disease, liver disease, kidney disease, thyroid disease, metabolic disease, or other lung diseases were excluded [12,13]. Therefore, 32 COPD patients with BMI less than 20 were included in this study. None of these patients were receiving nutritional support therapy or taking any medication at the time of evaluation. 22 aged and gender-matched healthy people from these communities, with normal physical examination and laboratory data and without medical illnesses, were enrolled as control group.

### Pulmonary function test

FVC and FEV_1_ were measured using standard spirometric techniques (MicroLoop; Micro Medical Ltd, Kent, UK). The test was performed according to ERS criteria [14]. The reference values employed were proposed by Zheng et al. [15].

### Body composition

Body height was determined to the nearest 0.5 cm with subjects standing barefoot. Body composition in this study was estimated using “foot-to-foot” bioelectrical impedance analysis (BIA) technology (QuickMedical, Snoqualmie, SE) while subjects were standing on blocks of solid metal. Lightweight, FM, Fat Free Mass, %fat and BMI were automatically calculated by the machine after age, height and gender were entered and standard calculation model was selected following manufacturer’s instructions.

### Sample collection

Fasting (from 9:00 p.m. of the previous night) blood samples was obtained by vein puncture at 8:00 a.m., centrifuged at 1,600 × g for 15 minutes at 4°C, The plasma was collected and stored at - 70°C.

### Obestatin assay

Obestatin was assayed with a sandwich enzyme linked immunosorbent assay kit (ELISA, Westang Bio-Tech Co. Ltd, Shanghai, China). The minimum detectable concentration was 100 pg/ml.

### TNF-α and C reactive protein (CRP) assays

Plasma TNF-α was measured using a high sensitivity ELISA (R&D, Minneapolis, MN, USA) according to the manufacturer’s procedure. The intra-and inter-assay variations were 6.0 and 7.5%, respectively. The minimum detectable concentration was 4.4 pg/ml. CRP in plasma was measured using an ELISA kit (Westang Bio-Tech Co. Ltd, Shanghai, China).

### Statistical analysis

Results are given as mean ± SD. Differences between the groups were statistically analyzed using an unpaired Student’s t test. Correlations were performed by bivariate Pearson correlation test. Significance was determined at the level of 5%. Data were analyzed with SPSS (Statistical Package for the Social Sciences, version 10.0 for Windows, SPSS Inc., Chicago, IL).

## Results

### Characteristics of the study subjects

Clinical characteristics of both the COPD patients and the control group are shown in Table 1. The COPD patients had airflow limitation and the control subjects had normal FEV_1_ values. BMI, waist circumference and hip circumference in the COPD group were lower than those in the control group. No significant differences were found regarding blood total cholesterol, triglyceride, creatinine, uric acid and blood urea nitrogen.

**Table 1:** Characteristics of the study subjects

	**Control**	**COPD**
Age, y	60.41 ± 6.60	59.78 ± 8.71
Sex, male/female	13/9	20/12
Smoking history, pack/year	12.96 ± 18.19	11.40 ± 16.39
BMI (kg/m2)	22.66 ± 2.96	18.99 ± 1.00*
FFM, kg	24.96 + 4.79	24.63 + 5.20*
Percent body fat, % weight	55.42 + 7.70	49.88 + 8.91*
Waist circumference (cm)	76.78 ± 9.64	68.13 ± 6.39*
Hip circumference (cm)	89.27 ± 7.12	84.54 ± 4.34*
Waist hip ratio	0.86 ± 0.06	0.81 ± 0.05*
FEV_1_/FVC, %	84.41 ± 4.58	63.66 ± 6.24*
FEV_1_, % predicted	85.51 + 20.23	69.81 + 15.99
Total cholesterol (mmol/L)	4.81 ± 1.16	4.68 ± 0.95
Triglyceride (mmol/L)	1.33 ± 0.53	1.04 ± 0.40
Creatinine (μmol/L)	88.32 ± 11.41	89.63 ± 16.78
Uric acid (μmol/L)	313.41 ± 68.12	302.31 ± 60.16
Blood urea nitrogen (μmol/L)	5.59 ± 1.33	5.72 ± 1.11

BMI, Body mass index; FFM, free fat mass; FEV_1_, forced expiratory volume in one second; FVC, forced vital capacity; COPD, chronic obstructive pulmonary disease. Values presented are mean ± SD. *indicate significant differences compared with normal control group (p < 0.05).

### Plasma obestatin in control group and COPD group

**Figure 1:**
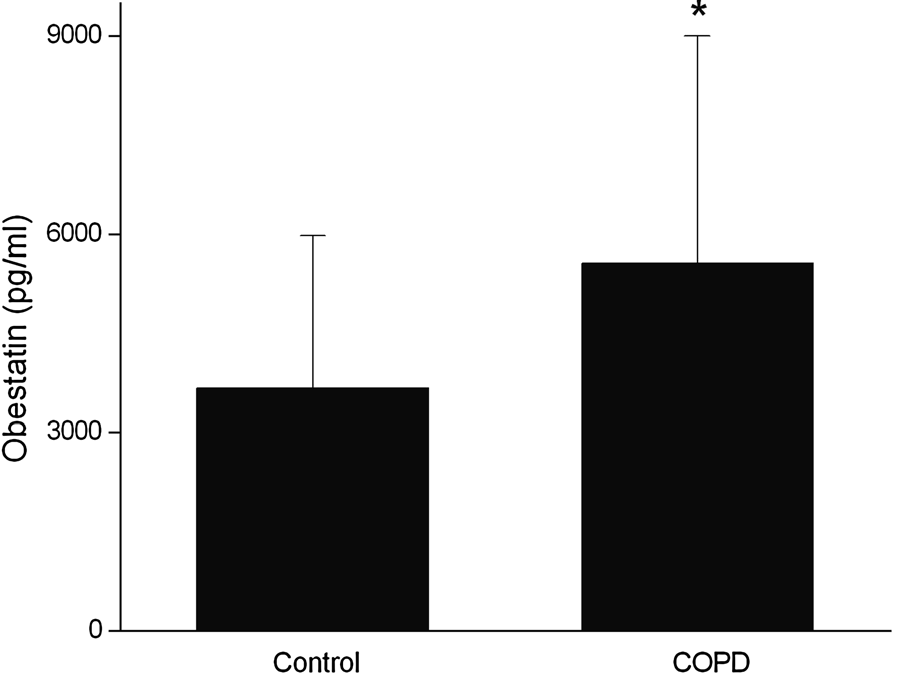
**Plasma obestatin level was significantly higher in COPD group than in age matched controls.** * indicate significant differences between control and COPD group (p < 0.05). COPD, chronic obstructive pulmonary disease.

Plasma obestatin was assayed by ELISA as described in Methods. As shown in Figure 1, the level of plasma obestatin was significantly higher in the COPD group than that in the age-matched controls (5562.75 ± 3435.43 pg/ml in COPD, 3663.90 ± 2313.95 pg/ml in controls, p = 0.028).

### Plasma obestatin does not correlate with BMI and other nutritional parameters

Treatment of rats with obestatin suppressed appetite, inhibited jejunal contraction, and decreased body-weight gain. We postulated that increased obestatin resulted in malnutrition in COPD, so we further investigated this trend. Correlation analysis was performed between obestatin and COPD nutrition status. However, bivariate correlate analysis did not show any significant correlation between the level of plasma obestatin and BMI, % body fat, FFM, waist circumference, hip circumference, and waist hip ratio. No significant correlation was found between obestatin and cholesterol, triglyceride, glucose, or insulin.

### Plasma obestatin correlates with systemic inflammatory markers

**Figure 2:**
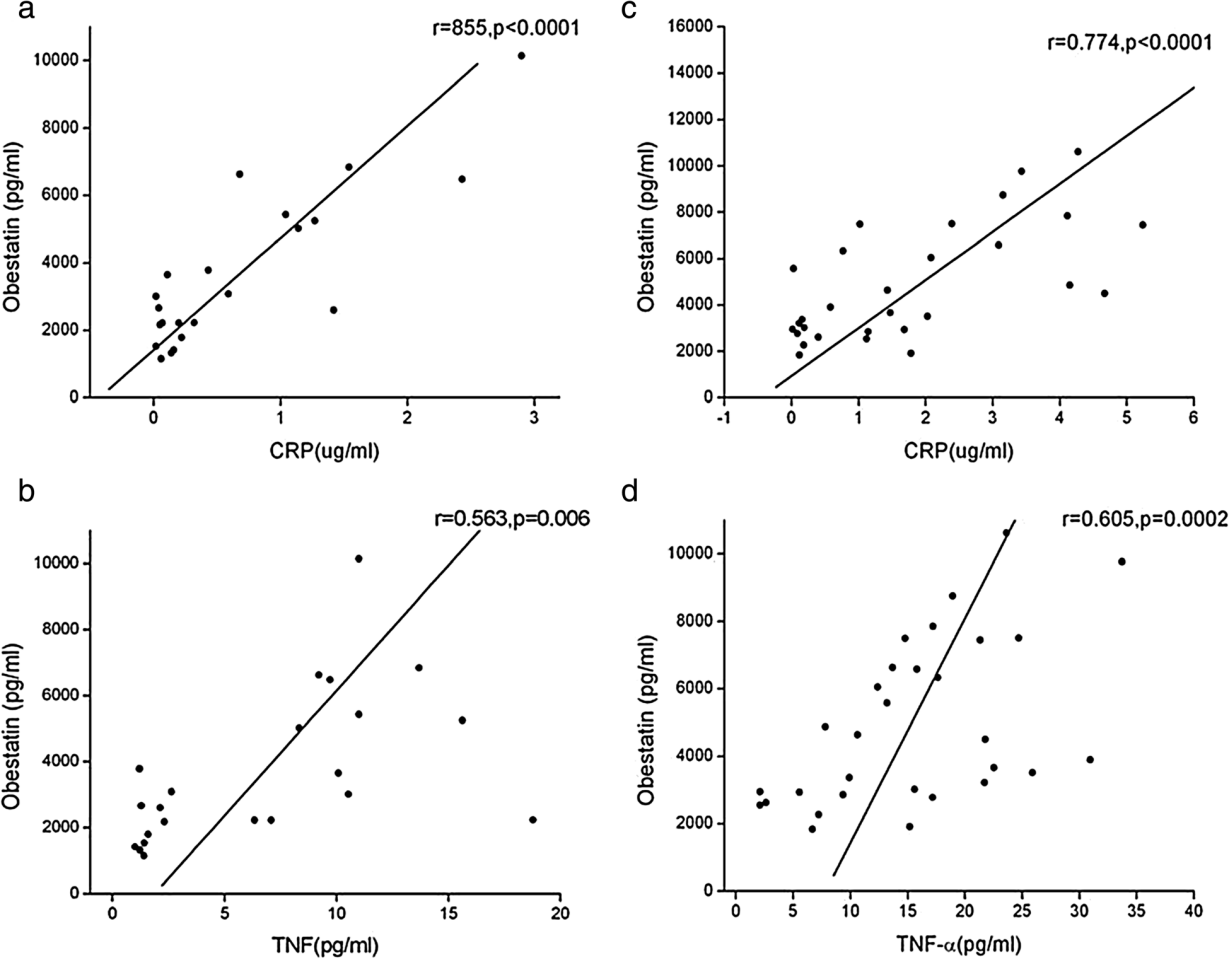
**Plasma obestatin correlates with circulating CRP and TNF-α (control group: Figure 2a and b. malnourished COPD patients: Figure 2c and d).** CRP, C-reactive protein; TNF, Tumour necrosis factor.

Systemic inflammation is a clinical characteristic of COPD. Previous researches showed that obestatin correlated with inflammatory markers in RA patients. Increased plasma obestatin may also correlate with inflammatory markers in COPD. As shown in Figure 2, plasma obestatin was positively correlated with the systemic inflammatory markers: CRP and TNF-α.

### Plasma obestatin does not correlate with pulmonary function in COPD

**Figure 3:**
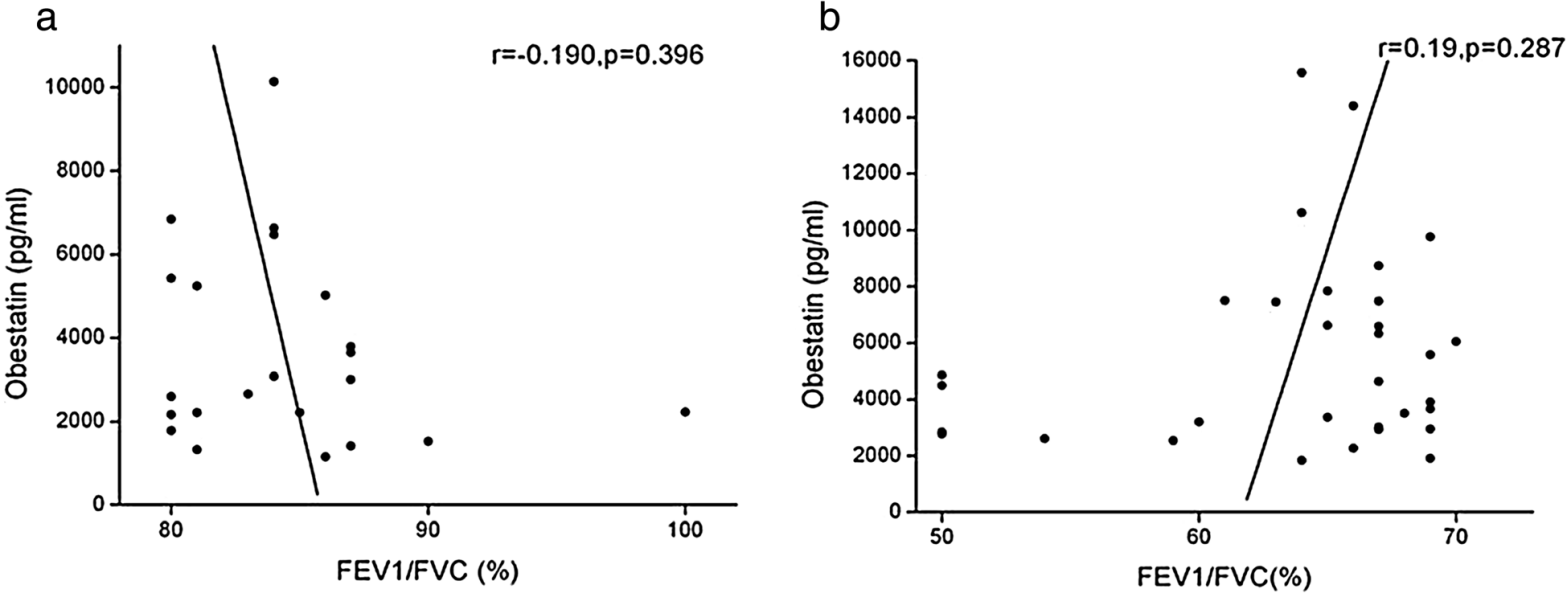
**Plasma obestatin does not correlate with FEV**_**1**_**/FVC% in control group (Figure 3a) and malnourished COPD patients (Figure 3b).** FEV_1_, forced expiratory volume in one second; FVC, forced vital capacity.

To further investigate whether the level of obestatin correlated with airflow limitation, bivariate correlation analysis was performed between the level of obestatin and FEV_1_/FVC%. As shown in Figure 3, the level of plasma obestatin does not correlate with this pulmonary function parameter.

## Discussion

In this study, we found that the level of plasma obestatin was significantly higher in COPD patients than in the control group. Significant correlation was found between the level of plasma obestatin and the systemic inflammatory markers: CRP and TNF-α. No significant correlation was found between the level of plasma obestatin and nutrition status parameters such as BMI, % body fat, FFM, waist circumference, hip circumference, waist hip ratio, cholesterol, triglyceride, glucose, or insulin. No significant correlation was found between the level of plasma obestatin and lung function.

Obestatin is a 23-amino acid peptide hormone released from the cells lining the stomach. Studies in humans have shown that plasma obestatin levels are significantly lower in obese subjects as compared to lean controls, indicating a role for obestatin in body weight regulation. Gao et al. [16] found the number of obestatin-positive cells in gastric body mucosa was significantly lower in overweight and obese patients than in healthy controls with normal weight. The concentration of plasma obestatin was also decreased in overweight and obese patients and positively correlated with the number of obestatin-positive cells in the gastric body mucosa. The authors suggested that overweight and obese subjects have a reduction in the number of obestatin-positive cells in the gastric body mucosa. In our study, the level of circulating obestatin was found to be higher in COPD patients with malnutrition than in the healthy control group. The exact mechanism of obestatin increase in COPD with malnutrition is not clear because the regulation of the expression of obestatin remains unknown at present. It was reported that the production of ghrelin, an adipokine derived from the same gene as obestatin, was regulated by other adipokines, (such as leptin) inflammation, or diet [17,18]. Zhang et al. [7] reported that although fasting led to higher level of ghrelin, the serum level of obestatin was constant after fasting in rats. These results suggested that although they are encoded by the same gene, the production of ghrelin and obestatin is regulated by different factors. Obestatin was found to be positively correlated with IL-6 in the BD patients, and with the level of TNF-α in the control group. It was also correlated with CRP and TNF-α in COPD. Increased obestatin may then be the result of inflammation in COPD, a disease with chronic systemic inflammation.

Obestatin was reported to suppress food intake, inhibit jejunal contraction, and decrease weight gain [7]. Although the level of plasma obestatin was higher in COPD patients compared with age-matched controls, no significant correlation was found between obestatin and BMI, % body fat, FFM, waist circumference, hip circumference, or waist hip ration in COPD. Although Xin et al. [13] reported that the level of obestatin was negatively correlated with BMI in chronic heart failure (CHF) patients, plasma obestatin did not correlate with BMI or other nutrition parameters in COPD in this study. Other studies also found that obestatin was inversely correlated with BMI in chronic kidney disease and hemodialysis patients. Gao et al. [16] reported that negative correlation was found between circulating obestatin levels and BMI in healthy subjects, but not in patients with chronic atrophic gastritis. This result suggests that obestatin expression in some diseases may be different from healthy patients. Zhang et al. [7] found that obestatin suppressed food intake in fasting mice and spontaneously reduced weight gain at large doses in lean mice. Another research demonstrated that obestatin exerted no effect on food intake and body weight in rats [19]. Other authors [14] found that Roux-en-Y gastric bypass (RYGB) surgery and/or the weight loss resulted in the decrease of ghrelin level but had no effect on obestatin levels. Thus, the role of obestatin in energy metabolism still remains uncertain.

Although the level of obestatin did not correlate with COPD nutritional status, correlation analysis showed that the level of plasma obestatin did positively correlate with CRP and TNF-α in COPD patients. It was reported that ghrelin inhibited various inflammation processes in arthritic rats, acute lung injury, and other inflammation diseases [20,21]. Since obestatin is derived from the same gene which also encodes ghrelin, it may play a role in systemic inflammation in COPD. Until now, only a few studies have investigated the role of obestatin in inflammation. Kellokoski et al. [10] reported that obestatin did not influence the adhesion of monocytes. Although obestatin treatment did not change the expression of either intercellular adhesion molecule-1 (ICAM-1) or monocyte chemoattractant protein-1 (MCP-1), obestatin treatment together with TNF-α suppressed the expression of VCAM-1, but did not alter ICAM-1 or MCP-1 expressions. As TNF-α and other pro-inflammatory cytokines increase in COPD, whether obestatin inhibits systemic inflammation by suppressing the expression of VCAM and other adhesion molecules requires further investigation. Koca et al. [22] reported that no positive correlation was found between the level of obestatin and some inflammatory markers in two chronic inflammatory diseases -RA and BD. This is different from our results. In their study, medications affecting the immune system were administrated to RA and BD patients but not to the control group. This may have affected the level of obestatin in their RA and BD groups. In contrast to our study in which no medication was administered to the COPD group or the control group, Xin et al. [13] reported that the level of obestatin was higher in CHF patients with cachexia compared with non-cachexia and normal controls. They also found that the level of plasma obestatin had a negative correlation with BMI. Since CHF is regarded as a disease with chronic systemic inflammation [23,24], the different levels of plasma obestatin in CHF patients and their negative correlation with BMI may be the result of the systemic inflammation of this disease.

## Conclusion

The level of circulating obestatin was higher in the COPD group compared with the control group. Obestatin was positively correlated with COPD systemic inflammation.

## Competing interests

The authors declare that they have no competing interests.
